# Abortion attitudes, training, and experience among medical students in Jamaica, West Indies

**DOI:** 10.1186/s40834-020-00106-9

**Published:** 2020-05-01

**Authors:** Glenmarie Matthews, Jessica Atrio, Horace Fletcher, Nathalie Medley, Leo Walker, Nerys Benfield

**Affiliations:** 1 Department of Obstetrics and Gynecology, Montefiore Hospital & Albert Einstein College of Medicine, 1695 Eastchester Road, Bronx, 10461 NY USA; 2 University of West Indies – Mona Campus, Kingston, West Indies Jamaica

**Keywords:** Abortion, Abortion attitude sum score (AbAs), Medical students, Abortion training, And Jamaica abortion

## Abstract

**Objective:**

To define abortion attitudes, training and experience among medical students in Jamaica, a restricted environment for legal abortion.

**Method:**

From September to November 2017 we conducted an anonymous online cross-sectional survey among medical students enrolled at the University of West Indies (UWI) in Jamaica. An abortion attitudes sum score was used for analysis. Multivariate regression was applied to evaluate the impact of characteristics and experiences on abortion attitudes.

**Results:**

The primary outcome was a validated composite abortion attitudes sum score, ranging from zero to forty-five. 1404 students completed the survey for a response rate of 88%. 64% had a positive attitude towards abortion. In multivariate analysis, medical students’ attitudes were favorably impacted by a prior personal or family experience with abortion, identifying as non-religious, being older in age and mixed raced. 1321 (94%) agreed that abortion training should be included in the medical school curriculum. 78.8% reported no abortion training and only 17.9% reported miscarriage management training.

**Conclusion:**

Medical students at UWI had favorable attitudes towards abortion, despite their limited training in a restrictive environment. Prior personal experience with abortion and being non-religious were the strongest predictor of favorable attitudes. Increased training and clinical exposure may prove to be crucial in improving access of safe abortion.

## Background

Unsafe abortion contributes to maternal mortality in the Caribbean and Latin America. These regions also have the highest estimated annual abortion rates in the world, at 65 per 1000 women of childbearing age in 2014 [1]. In Jamaica, abortion is legally restricted and unsafe abortion is prevalent. According to the World Health Organization more than 22,000 abortions occurred in Jamaica in 2011 and complications ranked as the eighth leading cause of maternal mortality in country [2].

The abortion law in Jamaica originates from the “Offences against the Person Act of 1861” modified by the decision in the Rex v. Bourne case of 1938 to permit abortion for health reasons [3, 4]. Current interpretation of the law cites that abortion is indicated to preserve the physical and mental health of the mother; but does not include rape or incest, fetal impairment, economic or social indications, or contraceptive failure [2]. In order to obtain a legal abortion two physicians must agree that the abortion is necessary. In the face of these restrictions, illegal or unsafe abortions are prevalent [5]. Previous research has demonstrated the majority of practicing Jamaican physicians (72%) felt that they did not have adequate training in abortion care [6]. However, the majority of Jamaican general practitioners (GPs) and obstetrician-gynecologists (obgyns) report there should be greater availability of induced abortions, which would reduce maternal mortality.

In settings where abortion is legally protected research among medical students has demonstrated favorable attitudes, which may in turn correlate with intentions to provide abortion care and advocate for women. A study of students at the University of Washington found that 70% supported legal abortion under any circumstances. Thirty-one percent intended to provide medical abortion in their practice, and 18% planned to offer surgical abortion [7]. A study in South Africa found that 70% of students believed women should have access to abortion [8]. Additionally, studies have found that abortion education, training, and care can influence student’s attitudes towards abortion [9–11].

There is no published data regarding the attitudes of Caribbean medical students towards abortion. Our research sought to assess attitudes, training, exposure, intentions to provide, and legal knowledge regarding abortion access and care among University of the West Indies (UWI) medical students in Kingston, Jamaica. UWI is the largest medical school in the Caribbean, with approximately 315 students enrolled annually, the majority of graduates practice in the Caribbean. The medical school consists of five years of training, the first three years are basic science course-work, and the last two years are clinical clerkships. Approximately 60% of graduates go on to complete one year of postgraduate internship before becoming general practitioners [12].

## Methods

From September to November 2017, we conducted an anonymous online cross-sectional survey of medical students enrolled at UWI. The UWI Institutional Review Board (IRB) and Einstein IRB approved the research. The UWI registrar’s office distributed electronic survey invitations and electronic consent was obtained. Research Electronic Database Capture (REDCap) was used to administer questions and compile data in an online portal. Duplicated questionaries’ where identified and deleted. As a descriptive survey, no power calculations were conducted and a sample of convenience was utilized.

The survey consisted of six domains: social and demographic characteristics, knowledge of the country’s abortion law, attitudes and beliefs about abortion provision, medical curriculum and training in abortion services, future abortion advocacy, and intention to provide abortion. The survey included questions from previously validated surveys, which have been described in the literature as well as new questions developed by the investigators [9]. The instrument was piloted among UWI faculty, and obgyn residents. In order to assess attitudes about abortion students were given a total of nine questions evaluating various reasons why a woman might seek and abortion. Student’s attitudes were scored from 1 to 5, for each scenario (eg. 1 = strongly disagree; 5 = strongly agree) [9]. An abortion attitudes sum score (AbAS) was calculated by totaling the responses, ranges of possible SS were 1 to 45. A favorable attitude towards abortion, was defined as an abortion attitudes sum score (AbAS) > or equal to 27.

Descriptive statistics and bivariate analyses were conducted using chi-square and t-tests or ANOVA, or their non-parametric equivalents. For analysis, clinical years of training were dichotomized into preclinical (years 1–3) vs. clinical (years 4–5), and intended specialty was divided into three categories - those who planned on being women health providers (obgyns and GPs), other specialties, and undecided. A multivariate linear regression model was developed in a stepwise fashion to identify associations between AbAS and individual characteristics. Any bivariate associations with the strength of association of at least *p* = 0.2 were assessed for inclusion in the multivariate model. Once an inclusive model was created, all contributing co-variates were assessed in a sequential fashion, if a variable no longer contributed equal to or greater than *p* < 0.05 it was removed. Once the model was finalized, assumptions of the multivariate linear regression model were evaluated and influential data (outliers) were removed.

## Results

Of the 1589 enrolled UWI medical students 1404 completed the survey for a response rate of 88%. The mean age was 22 and the majority identified as being single (61.9%), with only 4.3% being married. 597 (43%) identified as female, and 21% chose not to identify their gender (Table 1). Majority of the students were of African/ Afro-Caribbean descent (44%) with second most common race being mixed (18.7%). 54.7% of participants identifying as very religious. The most common religion was Christianity (72%) and 75% of students reported regular attendance at religious services. 71% of students reported personal exposure or knowing someone who have had an abortion. Majority of students intended to practice medicine in Jamaica (53.7%) or surrounding Caribbean nation (41.9%).

**Table 1:** Summary of Characteristics of Medical Students at University of West Indies (*n* = 1404)

Characteristics	N(%)
Age – mean (SD)	22.68 (2.87)
Gender	
Female	597 (42.52)
Male	509 (36.25)
Choose not to identify	298 (21.23)
Race	
Black/Afro Caribbean	621 (44.23)
Mixed	262 (18.66)
Other ^a^	521 (37.11)
Martial status	
Single	858 (61.99)
Married	59 (4.26)
Partnered	467 (33.74)
Years of training	
Preclinical	892 (63.53)
Clinical	512 (36.47)
Intended Specialty	
Women health provider	345 (24.57)
Other specialties	793 (56.48)
Unsure	266 (18.95)
Intended place of practice	
Jamaica	755 (53.77)
Caribbean nation	589 (41.95)
Other ^b^	60 (4.27)
Have you had or someone close to you had an abortion?	
Yes	998 (71.13)
No	405 (28.87)
Religion	
Christianity	1008 (72.31)
Other religions^c^	386 (27.69)
How often do you attend religious services?	
Never	344 (24.50)
≥ 1-2x a month	1060 (75.5)

^a^ Other races included - East Indian (91), Hispanic (3), Asian (83), choose not to identify (303)

^b^ Other intended place of practice – North America (52), Europe & United Kingdom (9)

^c^ Other religion - Hindu, Buddhist, Islam, Atheist, undecided

The mean AbAS was 32, and the majority of participants believed abortion should be legal (88%). 74% agreed that abortion should be legal for rape or incest, 94% if pregnancy was a threat to a women’s physical health, 91% if it was a threat to a women’s mental health, 91% for fetal anomalies and 91% for a woman recently infected with zika virus (Table 2).

**Table 2:** University of the West Indies Medical Student Attitudes Towards Abortion

	*N* = 1404	Agree N(%)	Disagree N(%)
Average Attitude Sum score (AbAS)^a^	31.59 ((±9.50)		
Would provide abortion in case of:			
Financial distress		677 (48.22)	727 (51.78)
Disruption of career or education goals		658 (46.90)	745 (53.10)
Family is complete		555 (39.56)	848 (60.44)
Rape/Incest		1039 (74)	365 (26)
Threat to maternal physical health		1326 (94.51)	77 (5.49)
Threat to maternal mental Health		1275 (91.01)	126 (8.99)
Preference for the different gender or fetal sex		456 (32.52)	946 (67.48)
Fetal anomalies		1274 (90.81)	129 (9.19)
Infection with Zika virus		1272 (90.66)	131 (9.34)

^a^ abortion attitude sum score (AbAS) ranges from 1 to 45, with ≥27 representing favorable attitude

The majority of participants reported limited training or exposure to abortion care. 78.8% stated that abortion care has never been included in any portion of medical school, with only 13.6% participating in the care of a woman who has had an abortion. Among the 297 (22%) of students who reported some abortion training, lecture was the primary means of education (96.9%) (Table 3). For the limited students who participated in direct patient care, it typically consisted of the medical abortion (85.3%). Majority of the students denied any exposure to or involvement in the care of a patient with an illegal abortion 1326 (94.7%).

**Table 3:** Training on abortion among University of the West Indies Medical Students

	N(%)
Has abortion care been included in any portion of your medical school?	
Not at all	1106 (78.83)
Somewhat	160 (11.40)
Sufficiently	137 (9.47)
Have you participated in abortion care during your training?	
Yes	192 (13.68)
No	1211 (86.32)
What experiences with abortion care have you had throughout your medical training ^a^	
Counseling on abortion	137 (71.73)
Medical abortion	163 (85.34)
Uterine aspiration	43 (22.51)
Management of complications	110 (57.59)
How many times have you participated in the care of a woman with a miscarriage	
Never	1148 (81.82)
> 1–5 times	255 (18.18)
How many times have you participated in the care of a woman who had a legal abortion	
Never	1329 (94.73)
> 1–5 times	74 (5.27)
How many times have you participated in the care of a woman who had an illegal abortion?	
Never	1326 (94.65)
> 1–5 times	75 (5.35)

*chi-square or fisher exact test

^a^ of the 13.6% of student that participated in abortion training

Knowledge of the abortion law in Jamaica was limited. Approximately 65% of participants were unable to accurately identify one of the reasons a woman could legally access a therapeutic abortion in Jamaica, which includes threat to mental or physical health of a women. Students in their clinical years were more likely to identify any of these indications (64.8%) compared to students in the preclinical years (40.9%) *p* < 0.001 (Table 4).

**Table 4:** Knowledge about Abortion among University of the West Indies Medical Students

	N(%)	Preclinical years	Clinical years	*P*
In Jamaica abortion in is?				< 0.001
Always penalized	457 (32.57)	351 (39.39)	106 (20.70)	
Legal for the mental or physical health of the woman^a^	381 (27.16)	168 (18.86)	213 (41.60)	
Legal if the pregnancy is the result of a rape	92 (6.56)	54 (6.06)	38 (7.422	
Legal for fetal anomalies	112 (7.98)	60 (6.73)	52 (10.16)	
Legal when the mother has a disease that can worsen with pregnancy ^a^	180 (12.83)	93 (10.44)	87 (16.99)	
Legal under certain circumstances ^a^	406 (28.94)	232 (26.04)	174 (33.98)	
Legal in all cases	3 (0.21)	2 (0.22)	1 (0.20)	
I do not know	248 (17.68)	175 (19.64)	73 (14.26)	

^a^ Indicates the reasons for which a woman can obtain a legal abortion in Jamaica

81% of students were willing to refer patients to other clinics and providers if a woman requested an abortion (Table 5). However, only 398 (28%) of medical students intended to incorporate medical or surgical abortion into their future practice. The most common reason for not intending to provide abortion was to not oppose the law (36.7%), and 659 (46%) of all medical students reported being willing to provide abortion if the legal restrictions were lifted. 345 (24.6%) of participants reported that they plan to practice as an obgyn or GP. Approximately half of these future women’s health providers intend to provide medical or surgical abortion care 159 (46%). 178 (52%) of future women’s health providers reported they would be willing to provide abortion care if the legal restrictions were changed.

**Table 5:** Intention to provide abortion by intended specialty among University of the West Indies Medical Students

	N (%)	Woman’s health provider^a^	Other specialties	Unsure	P
Intention to provide medical or surgical abortion care					< 0.001
Yes	398 (28.35)	159 (46.09)	151 (19.04)	88 (33.08)	
No	740 (52.71)	132 (38.26)	474 (59.77)	134 (50.38)	
I don’t know	266 (18.95)	54 (15.65)	168 (21.19)	44 (16.54)	
Would you be willing to refer patient for abortion services					0.004
Yes	1134 (81)	257 (74.49)	663 (83.92)	214 (80.75)	
No	162 (11.57)	57 (16.52)	77 (9.57)	28 (10.57)	
I don’t know	104 (7.43)	31 (8.99)	50 (6.33)	23 (8.68)	
Would you be willing to provide abortion if the legal restrictions were lifted					0.078
Yes	659 (47.04)	178 (51.59)	352 (44.50)	129 (48.68)	
No	469 (33.48)	114 (33.04)	276 (34.89)	79 (29.81)	
I don’t know	273 (19.49)	53 (15.36)	163 (20.61)	57 (21.51)	

^a^Women health providers include obgyn & family medicine

The majority of respondents (*N* = 1113, 80%) agree that they would be willing to advocate for women’s health. However less than half of respondents (41%) stated they would be willing to advocate for abortion access and care in Jamaica. Slightly more students who intend to be women health providers are willing to advocate for change to the law to expand access to abortion care: 33% compared to 26% of persons pursuing other health specialties, *p* < 0.001.

The development of the model with sequential stepwise analysis of the cohort resulted in a more parsimonious model, which included a total of 13 characteristics. In the multivariate regression model co-variates that favorably impacted abortion attitudes sum score, included identifying as mixed raced, older age, not religious or reporting a personal or family history experience with abortion (Table 6). Covariates correlated with a lower score, and a more negative abortion attitude, included participants who identified as Afro-Caribbean, single, inaccurate knowledge of the abortion law, lack of abortion training, refusing to refer a patient for abortion care and a belief that abortion should not be legal.

**Table 6:** Multivariate Logistic Regression Model Regarding Abortion Attitude Sum Score and Associated Characteristics among University of the West Indies Medical Students

Dependent Variable	Independent variables with favorable impact	***β*** coefficient (95% CI)	*P* value
Attitude			
	History of personal or family exposure to abortion	2.63 (1.92 to 3.34)	< 0.001
	I am not religious	2.74 (2.09 to 3.38)	< 0.001
	Race (Mixed)	2.58 (1.69 to 3.47)	< 0.001
	Would be willing to provide abortion if law was lifted	0.99 (0.34 to 1.66)	0.003
	Intend to become women health provider^s^	0.86 (0.12 to 1.60)	0.022
**Dependent Variable**	**Independent variables with negative impact**	*β***coefficient (95% CI)**	***P*value**
Attitude	Abortion should not be legal in Jamaica	−15.65(−16.93 to −14.37)	< 0.001
	Inaccurate knowledge of abortion law	−5.60(−6.30 to −4.89)	< 0.001
	Afro-Caribbean Race	− 4.27(− 5.11 to −3.45)	< 0.001
	Not willing to refer patient for abortion	−3.85(− 4.95 to −2.74)	< 0.001
	No training in abortion	− 2.78(− 3.67 to − 1.89)	< 0.001
	*Martial status (single)*	−1.47(− 2.17 to - 0.77)	< 0.001
	Female gender	- 0.40(− 1.02 to 0.23)	0.22
	Older age (years)	−0.25(−0.39 to −0.13)	< 0.001

^a^Women health provider = General Practitioner and obstetric& gynecology

## Discussion

The majority of UWI medical students report favorable attitudes towards abortion in the setting of nationally restricted abortion access, and majority stated that abortion should be legal in Jamaica. These attitudes are comparable to those reported by medical students in the United States, where abortion is federally protected [13]. Internationally, favorable attitudes toward abortion have been noted among medical trainees at comparable levels in South Africa (70%), where abortion laws have changed in the last 22 years to be legally permissible for all indications up to 12 weeks [8]. Furthermore, acceptance and attitudes of students are comparable to the attitudes reported by Jamaican general practitioners surveyed in 2008 [6, 8, 14].

Comparable to previous research among general practitioners, current students report limited or absent exposure to or training in abortion care [6]. If students reported any training is was predominately in lecture format. This may not be the optimal format to engage in abortion education; pervious research among trainees and practitioners demonstrated that they would prefer, and stand to gain more impactful knowledge from, clinically applied and patient focused abortion care and management of complications through applied experiences [15].

The minority of students reported accurate knowledge of the legal status of abortion in Jamaica. Additionally, a third of participants though abortion was effectively illegal under any circumstances (including a threat to a mother’s life) and subject to persecution in Jamaica, with the concomitant concern that physician are penalized and incarcerated for participating in abortion care (32.5%). Consistent with these perceptions the majority of respondents stated that they would not be willing to provide abortions for fear of violating the law. The law serves as a tremendous deterrent to provision, and effectively is a means to re-stigmatize reproductive health care, by causing reproductive health providers to make practice decisions and medical decisions based on a potentially flawed and imperfect understanding of the law. Erroneous knowledge of legal restrictions, with concern for possible incarceration could be a real contributor to inadequate access to abortion care, inadequate management of complications, or lack of referral to abortion care in a country that experiences tremendous maternal morbidity from unsafe abortion.

Opportunities for improved educational experiences or values clarification workshops on abortion and abortion training could impact student’s readiness and willingness to engage in abortion related care. Furthermore education regarding the current legal indications for abortion may demystify, destigmitize, and decriminalize the scope of practice and engage students with evidence based, lifesaving, translatable skills related to abortion care as well as the medical management of septic abortion or miscarriage. Furthmore, students report that Jamaican women should have access to abortion through revision and changes in the laws. The majority of respondents have personal experience with abortion and the majority consider themselves to be women’s health advocates which also harnesses the potential for this community to implement effective women’s health policies and evidence-based care practices for the women of the Caribbean.

Religion is a potentially fixed and non-modifiable identity characteristic associated with medical professional abortion-related attitudes [8]. Previous research have suggested that health care providers recognize the distinction between personal and professional attitudes toward abortion, and believe that a personal religious objection does not impact on the provision of legal services to women, including nondirective counseling, referrals, and surgical and medication abortion [16, 17]. By contrast our research suggest that personal values impact attitudes and potentially practice because non- religious students (45.5%) had more favorable attitudes towards abortion (*p* < 0.001). Other research has demonstrated that personal experience and exposure to abortion impacts beliefs & perceptions, whereby “a broader understanding of the vagaries of existence that make abortions at times unavoidable” can impact attitudes toward abortion [8]. Our data demonstrate that personal experience with and or a family exposure to abortion were the most powerful and significant contributors to a favorable AbAS.

### Strength

The strength of our research was the robust response rate, which in turn improved the fitness of our analysis and model development. We are confident that our results accurately represent the UWI medical student community, which comprise a diverse and international student body. We consider our findings to be representative of the attitudes of future Jamaican physicians and the surrounding West Indian Islands. We achieved a high level of response through the engagement of faculty and students, integration of the registrar and a collaborative stakeholder approach. Recruitment of medical students was done directly through course lectures, and other peer student’s recruitment. An additional strength is the instrument itself, which was designed carefully, piloted extensively, and tested for internal reliability and consistency.

### Limitation

As a survey this instrument was subjected to limitations related to self-reporting and recall bias. Researchers did not have the opportunity to longitudinally assess participants over time to see if these intentions and attitudes are reflective of practice patterns and abortion provision. Additionally, social desirability bias may have influenced people to identify more favourable intentions regarding reproductive rights. However, the confidential and de-identified nature of the survey was intended to minimize these concerns.

## Conclusion

Medical students had favorable attitudes towards abortion. However, training in and exposure to abortion are extremely limited. Having prior personal experience with abortion was the most impactful predictor of favorable attitudes. Modifiable characteristics like training during medical school and an awareness of the current legal indications for abortion are opportunities to improve medical students awareness and attitudes regarding the public health impact abortion has on the women of Jamaica. These modifiable changes can combat unsafe abortion maternal mortality.

## Data Availability

The dataset used and/or analyzed during the current study are available from the corresponding author on reasonable request.

## Abbreviations

AbAs: Abortion attitude sum score
GPs: General practitioners
obgyns: Obstetrician-gynecologists
UWI: University of the West Indies
IRB: UWI Institutional Review Board
REDCap: Research Electronic Database Capture
